# Turpentine in Large Doses in the Treatment of Purpura Hæmorrhagica

**Published:** 1846-01

**Authors:** J. Moore Neligan


*Turpentine in Large Doses in the Treatment of Purpura Hæmorrhagica.* By J. Moore Neligan, M. D., M. R. I. A.—It is now very generally admitted that there is not the least similarity, either in their nature or origin, between purpura hæmorrhagica and the scurvy of seamen. Nevertheless, this idea, the correctness of which was so strongly insisted on by Willan, still influences much the opinions of many practitioners, with reference to the treatment of this disease; and the statement put forward by our great English authority on skin diseases, "that the treatment of this disease is simple, and may be comprised in a very few words: a generous diet, the use of wine, Peruvian bark, and acids," is, even in the present day, too indiscriminately adopted On the other hand, we find it laid down by numerous writers on the disease, who adopt the opinion of Dr. Parry, *that it is always of inflammatory origin*, that early and free venesection alone holds out any hope of successful treatment. My intention in the present communication is, without attempting to reconcile or account for those conflicting opinions, to narrate some cases of purpura which were speedily and effectually cured by the administration of turpentine in *large doses*, and at the same time to state the reasons which first led me to employ it.

In the ninth volume of the *Edinburgh Medical and Surgical Journal*, Dr. Harty of this city, in a communication to Dr. Bateman of London, details some cases of purpura simplex, and of pupura hæmorrhagica, in which the free employment of purgatives was attended with marked and rapid success. The purgatives employed by him were calomel and jalap; and he states that he was induced to adopt this mode of treatment of purpura from incidental remarks of the good effects of purgatives in his disease, to be met with in in the writings of Heberden, Hoffman and others. In the spring of 1840, while acting for a few months as one of the physicians of the city of Cork Dispensary, I met with eight cases of purpura hæmorrhagica of the worst form. The district which I had the charge of (Blarney-lane and its neighbourhood) being one of the poorest in the city, the individuals who were attacked with the disease were nearly all of broken-down constitutions, owing to outwork and insufficient nutriment. Having, in consequence of the asthenic character of the disease, treated the first two cases which came under my care, on the tonic plan, without success; in the next case I met with I had recourse to the employment of free purgation, but this case, which, however, was not seen until the disease was very far advanced, also terminated fatally. The fourth case, in which the individual was younger and of a more robust habit of body, terminated favourably under the free use of purgatives, employed as directed by Dr. Harty.

From the result of these four cases I was, of course, led to place but little reliance on the use of bark and acids in the treatment of this disease, and to look more favourably upon the employment of purgatives. I thought, however, that still more favourable results might be expected from the administration of oil of turpentine, which, while it acts as a powerful cathartic, also possesses the property of checking hæmorrhage, depending on an atonic state of the smaller blood-vessels, owing, probably, to its powers as a diffusible stimulant. In consequence of those views, I employed this remedy in the four cases that afterwards came under my care while in charge of the district, and they all recovered. I prescribed the oil both in the form of draught and of enema; the usual dose for adults being from one ounce to an ounce and a half, and for children from two drachms to half an ounce, generally in combination with castor oil, to render its carthartic action more certain.

Since that time I have employed oil of turpentine in every case of purpura which has been under my care, and its use has been invariably attended with beneficial results. The mode of its administration, and the effects which it produces, will be better understood from a perusal of the following cases, the two first of which I have selected as being evidences of the effects of the remedy, both in the child and in the adult, and also as having been witnessed by the clinical class in the hospital; and the third, in consequence of its having been attended, in consultation, with an intelligent practitioner of this city, who was at first adverse to the use of the oil of turpentine in such large doses.

Case I. Reported by Dr. J. O. Curran.—*Sore Throat, Anorexia, and general Depression of a Patient exposed to Contagion of Scarlatina; Occurrence of Purpura Hæmorrhagica four days after; Turpentine per Os et Anum in large doses; rapid and uniform recovery.*—Anna Welby, a remarkably fine-looking child, six years of age, was admitted into Jervis-street Hospital on the 11th of April, 1843. She is robust, but very pale, and her countenance has a most languid and anxious expression; the lips and nostrils are covered with blood of a dark colour, which has coagulated over them, and blood is oozing slowly from the margins of the gums; an eruption of small circular spots, about two lines in diameter, and varying in colour from a blackish purple to the hue of arterial blood, is thinly and pretty uniformly diffused over the whole body; the spots are nearly all of the same size, and perfectly circular, but a few closely resemble vibices both in colour and outline; the colour of the eruption is not in the least affected by pressure, nor by the part of the body in which it occurs; a few spots are sensibly prominent, and there are also some which are mere bloody vesicles, and which rupture under slight pressure with the nail; one or two spots are situated on the red margin of the lips, as well as on the mucous membrane of the mouth; the tongue is moist and slightly furred, and the papillæ, which are red and prominent, give it a mottled appearance; the fauces are very red, and the right tonsil considerably enlarged, puckered, and of a deep brownish-red colour; the pulse 120, small and hard; the respiration quiet, and there is no cough or expectoration.

The history of the case is shortly as follows:—patient slept in the bed with her brother and sister, who had just been attacked with scarlatina. On Thursday (the 6th) she was observed to change colour several times, she abandoned play, and could not be induced to eat anything. The following day she complained of her throat being very sore, and her mother observed that it was swollen; sickness was also complained of. The next day there was no alteration. On Sunday morning the eruption was first perceived; her gums were then bleeding, and in the course of the day she passed blood by urine, by stool, and also by vomiting; she had also several attacks of epistaxis, which, however, were very slight, and subsided spontaneously; on the next evening she was admitted in the hospital.

*April 12th.* Was very restless during the night, and could not be induced either to eat or drink anything; slept little; this morning her countenance has the same appearance of languor, there is more depression, but the pulse, &c., continue as before; the patient will not answer questions, nor even open her mouth, or put out her tongue when desired to do so.

Many new spots have made their appearance; they are of a florid-red colour, whilst the hue of those previously noticed has become darker; no new vibices have been observed, and there has been no epistaxis since her admission; blood still oozes from the gums, and occasionally from the nares, which the patient is continually irritating with her fingers; the urine is said to have been of a porter colour, but it was not preserved; the bowels have not acted since her admission. ℞. Olei terebinthinæ, olei ricini, utriusque, ʒiii, Aquæ menthæ piperitæ ℥ss. Misce, Fiat haustus statim sumendus, et vespere, ni alvus prius responderit, repetatur.

*April 13th.* The above draught was given twice, but it speedily excited vomiting; the whole of the medicine, however, did not appear to have been ejected from the stomach; it had no action whatever on the bowels, and consequently five grains of calomel, with an equal quantity of scammony, were administered at bed-time, by the directions of the house-surgeon.

The eruption is unchanged ; the skin is hot ; the pulse hard, and ranging about 130 ; the tongue has lost the mottled appearance which it presented on the day of admission, but it is still slightly furred ; the fauces are red, but the swelling of the right tonsil is diminished.

The bowels have not been moved ; there is no pain complained of, the respiration is but very slightly accelerated, and there is no cough whatever. ℞. Olei terebinthinæ, olei ricini, *aa* ℥ss, decocti hordei, ℥x, Fiat enema, statim adhibeatur.

*April* 14*th.* The injection operated freely, bringing away a considerable quantity of feculent matter, intimately mixed up with grumous blood.

The improvement in the appearance of the patient is of the most marked and decided character. The countenance has partially regained its colour and animation, and the patient is sitting up in bed, amusing herself, and readily answering questions. The pulse is less frequent and not so hard ; the tongue quite clean and moist ; the skin cool. No new spots have made their appearance, and those which were previously present have become much darker-coloured. ℞. Olei terebinthinæ, olei ricini, *aa* ʒii. decocti hordei, ℥x, Fiat enema, hodie injiciendum.

*April* 15*th.* Continues to improve. The enema to be repeated.

*April* 16*th.* Dejections still consist almost wholly of grumous blood, but mixed with a much larger and very evident proportion of feculent matter. Pulse 120 ; respirations 24 ; skin of natural temperature ; eruption much faded ; expression of countenance cheerful and healthy. The enema to be repeated.

*April* 17*th.* Had two perfectly natural dejections after the enema ; feels and looks quite well : spots very much faded.

*April* 20*th.* Countenance quite healthy and lively ; eruption scarcely perceptible. The bowels being confined, she was ordered a mild purgative of calomel and scammony.

*April* 24*th.* Discharged cured.

This child was admitted into hospital again on the 2d of January, 1845, nearly two years afterwards, labouring under a second attack of purpura, not nearly so severe, however, as in the first instance. The oil of turpentine was administered to her in the form of draught, uncombined with castor oil, the quantity prescribed being two drachms, night and morning, for five successive days ; it was given floating on the surface of peppermint water, in which form it was retained by the stomach, and produced from three to four stools daily. She was quite well on the 7th instant, the sixth day after the appearance of the spots, but she was kept in the hospital until the 12th of January, for fear of a relapse.

Case II. *Purpura Hæmorrhagica, occurring in an adult, cured by large doses of Oil of Turpentine.* Reported by Mr. Farmer.—William Flannagan, aged 50, a labourer, admitted into Jervis-street Hospital, July 1, 1845. The entire of the body and limbs is covered with small circular spots, of various size and colour ; from half a line to a line in diameter. and varying in colour from the florid red of arterial blood to a purplish-black hue. There are also several large, ecchymosed patches of a deep greenish-purple colour; those are situated chiefly on the right mamma, the elbows, the loins, and the backs of both legs. Firm pressure produces no effect on either the small or large spots. He complains very much of weakness, with pain in his back, which, together with a feeling of great lassitude, has, from the commencement of his illness, altogether prevented him from working. He is constantly coughing up a frothy serum, deeply tinged with blood; the gums also bled slightly, and he states that, previous to his admission into hospital, he passed bloody stools. The pulse beats about 60 in the minute, but is feeble and very compressible. The body is emaciated, and the countenance very expressive of anxiety.

In early life the patient was addicted to intemperance, nevertheless he enjoyed perfect health until the first attack of the present disease, which was about six months ago. Since that time he has been repeatedly attacked with the disease, but at no time so severely as at the present. He was in a hospital during the first seizure, where he was cured of it, but it reappeared in three months afterwards; he was again admitted into the same hospital, but having been discharged before the spots completely disappeared, they in a few days began to increase in size and in number, and he has never been free from them since. The great size of the vibices, together with the bloody dejections and sputa, and the complete prostration both of mind and body, compelled him at length to seek admission into this hospital.

*July 2d.* Many new spots have made their appearance since yesterday, and the bowels have not been moved since his admission.—℞. Olei terebinthinæ ℥iss, syrupi ℥ii, aquæ menthæ piperitæ ℥ii. Misce, Fiat haustus statim sumendus.

*July 3d.* Was somewbat intoxicated yesterday after taking the draught, which vomited and purged him freely, the stools being slightly mixed with grumous blood. He feels much better to-day, and eats with an appetite, which he has not done for some time. The spots are darker colored than on admission, and some new ones have made their appearance, but the sputa are not so bloody.

*July 4th.* The large blotches are fading, and turning of a yellowish green colour, while the small spots are disappearing; sputa still tinged with blood; bowels not moved yesterday.—℞. Olei terebinthinæ ℥iss, olei lini ℥i, decocti hordei ℥xvi. Fiat enema, et statim adhibeatur.

*July 5th.* The patient is improved in every respect, with the exception of the sputa, which are more bloody; the bowels were affected only once by the enema; there is no appearance of blood in what he passed.—℞. Olei terebinthinæ ℥i; syrupi ℥ss; aquæ menthæ piperitæ ℥ii; Misce: Fiat haustus, statim sumendus.

*July 7th.* Still improving; both large and small spots are gradually disappearing; bowels rather confined. The draught to be repeated, and to have full diet.

*July 9th.* Feels quite well to-day; none of the small spots to be seen, and the larger blotches much diminished in size ; has had no expectoration for the last two days; as the bowels were confined, he was ordered the common castor oil draught.

*July* 12*th.* Flanagan was discharged to-day quite cured, having been kept in hospital until all the stains disappeared from the skin.

The third case was that of a delicate child, five years of age, whom I attended in consultation wtth my friend Mr. Dobbyn, of D'Olier Street, in May, 1843. After two days slight fever, the entire body became, in one night, thickly covered with spots of purpura, while two large vibices were apparent on the nares, evidently produced by the pressure of the body on that part ; the bowels were free, but the stools consisted of feculent matter, intimately mixed with blood. The oil of terpentine was administered to her in the form of draught, in doses of two drachms and a half twice daily. She was only five days confined to bed, and on the sixth day scarcely a trace of the disease could be perceived on any part of the body.

This case I look on as being particularly interesting, when considered in connexion with that of Welby, the first case I have related in this communication, inasmuch as this was an exceedingly delicate child, of a rather strumous habit of body, while the girl Welby was a fine, healthy-looking child, with, after her recovery, a very florid complexion. It thus appears, that this mode of treating the disease is equally applicable when it occurs in the robust as in the debilitated—a fact which is fully born out by the experience I have had of it for the last five years.—*Lond. and Ed. Monthly Journal.*

